# Comparative Risk of Cancer Recurrence During Treatment With Biologics: A Meta-Analysis

**DOI:** 10.1016/j.gastha.2026.101092

**Published:** 2026-08-15

**Authors:** Daniel Isufi, Christopher Willy Schwarz, Mikkel Bak Jensen, Jakob Seidelin, Lone Skov, Nikolai Loft

**Affiliations:** 1 Department of Dermatology and Allergy, Copenhagen University Hospital - Herlev and Gentofte, Copenhagen, Denmark; 2 Copenhagen Research Group for Inflammatory Skin, Herlev and Gentofte Hospital, Gentofte, Denmark; 3 Department of Clinical Medicine, Faculty of Health and Medical Sciences, University of Copenhagen, Copenhagen, Denmark; 4 National Allergy Research Centre, Department of Dermatology and Allergy, Herlev and Gentofte Hospital, Gentofte, Denmark; 5 Department of Gastroenterology and Hepatology, IBD Section, Herlev and Gentofte Hospital, Herlev, Denmark

Patients with immune-mediated inflammatory diseases (IMIDs) and a history of cancer pose a therapeutic challenge, as the safety of biologic therapies in this context remains uncertain. Indeed, the labels of biologics for IMID state caution in patients with malignancy, and patients with a recent malignancy were excluded in the pivotal trials of these drugs. Recently, we found no increased risk of cancer recurrence comparing biologics with conventional systemics.^1^ However, studies comparing biologics with conventional systemics are at risk of healthy initiator bias, why studies comparing biologics are urgently needed. Therefore, we conducted a systematic review and meta-analysis to compare the risk of new or recurrent cancer in patients with IMIDs and a history of malignancy treated with biologics, applying the same methodology as a recent study.^1^ In brief, risk estimates were calculated with random-effects meta-analysis with I2 statistics to estimate between-study heterogeneity.

In total, 16,838 records were identified (PubMed = 2,280, Web of Science = 3,635, Embase = 9,922, and Cochrane Library = 1001) with inception through September 28, 2025. Overall, 4 studies^2^, ^3^, ^4^, ^5^ were included encompassing 676 patients (tumor necrosis factor alpha inhibitor [TNFi] = 402, vedolizumab = 218, and ustekinumab = 56) who developed 121 cancers (TNFi = 86, vedolizumab = 28, ustekinumab = 7). All studies were conducted in inflammatory bowel disease (IBD) (Supplementary Table 1).

Meta-analyses could be conducted for new and/or recurrence of any cancer for TNFi vs interleukin (IL)-12/23i, TNFi vs anti-α4β7-integrin, and IL-12/23i vs anti-α4β7-integrin.

For recurrence of any cancer for TNFi vs anti-α4β7-integrin, the risk ratio (RR) was 1.47 (95% confidence interval [CI], 0.77-2.78; *P* = .12) based on 3 studies^2^, ^3^, ^4^ comparing 324 patients treated with TNFi with 38 recurrent cancers to 167 patients treated with anti-α4β7-integrin with 14 recurrent cancers. The corresponding incidence rate ratio (IRR) was 1.56 (95% CI, 0.77-3.19; *P* = .11) comparing 2703 patient-years (PYs) of TNFi and 38 recurrent cancers to 1396 PYs of anti-α4β7-integrin and 14 recurrent cancers (Figures 1 and 2, and Supplementary Table 2).

**Figure 1:**
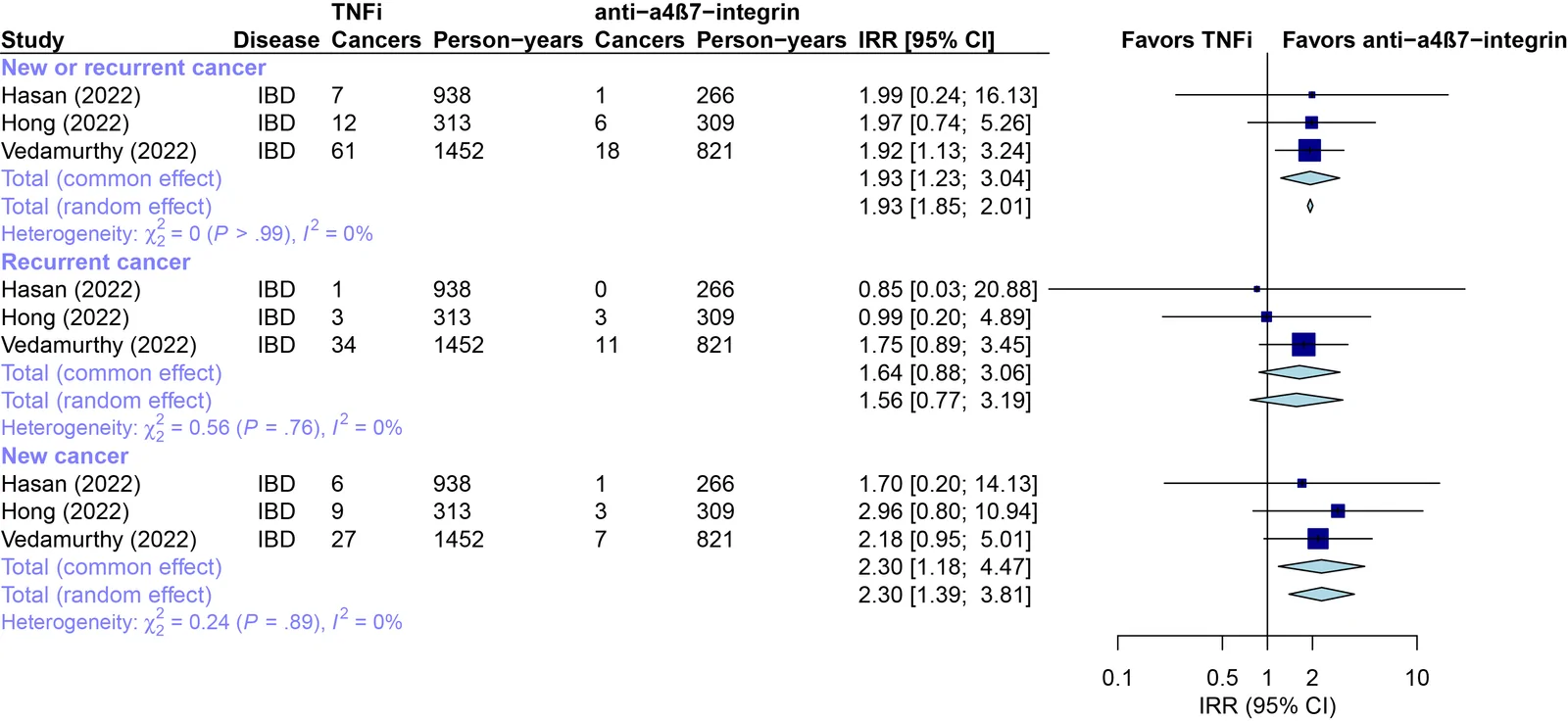
Forest plot of incidence rate ratio of TNFi compared to anti-α4β7-integrin.

**Figure 2:**
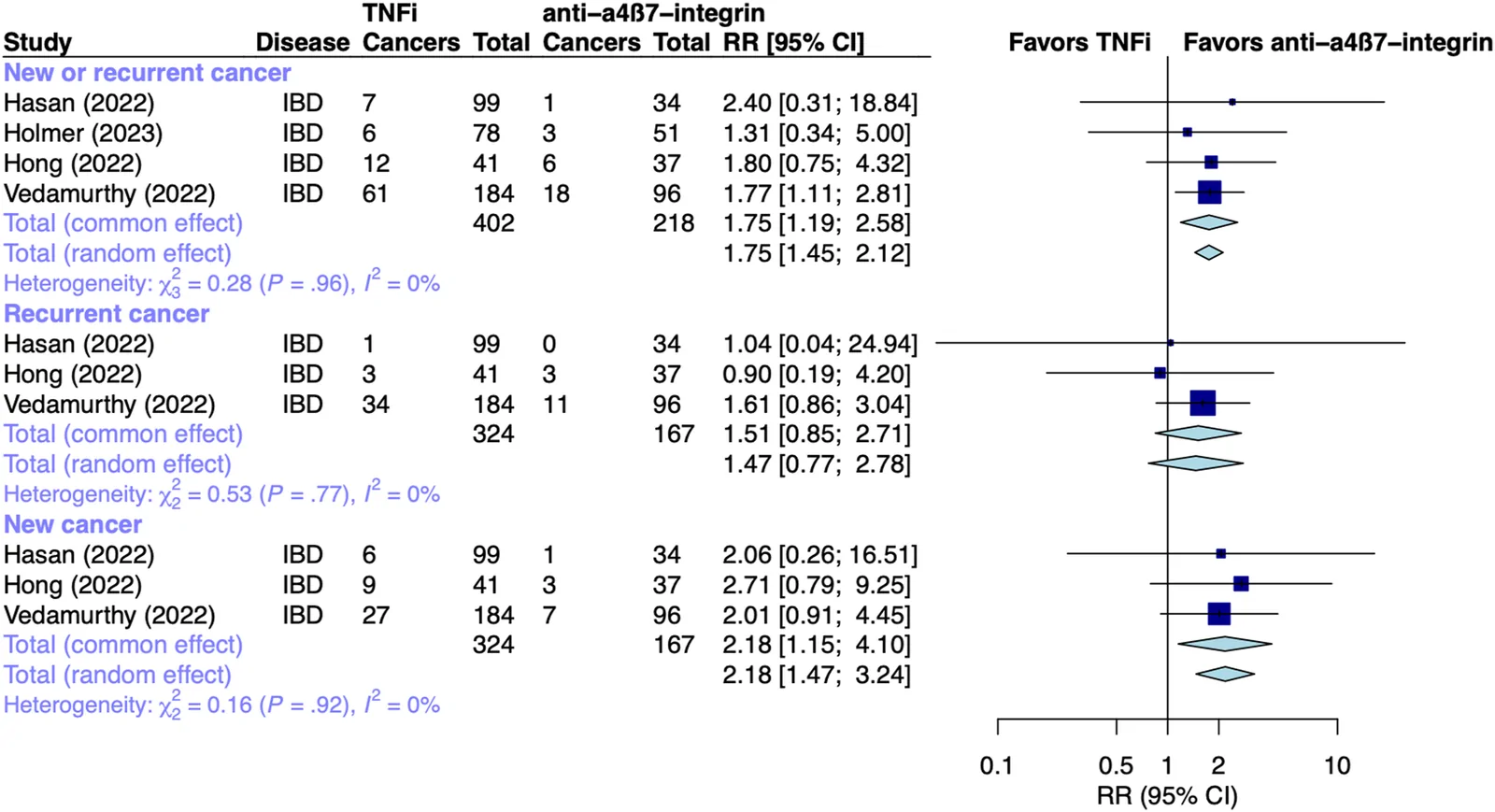
Forest plot of risk ratio of TNFi compared to anti-α4β7-integrin.

For any new cancer for TNFi vs anti-α4β7-integrin, the RR was 2.18 (95% CI, 1.47-3.24; *P* = .013) based on 3 studies^2^, ^3^, ^4^ comparing 324 TNFi-treated patients developing 42 new cancers to 167 anti-α4β7-integrin-treated patients developing 11 new cancers. The IRR was 2.30 (95% CI, 1.39-3.81; *P* = .019) based on 2703 PYs and 42 new cancers in the TNFi group, and 1396 PYs and 11 cancers in the anti-α4β7-integrin group^2^, ^3^, ^4^ (Figures 1 and 2, and Supplementary Table 2).

For new or recurrent cancers for TNFi vs anti-α4β7-integrin, the RR was 1.75 (95% CI, 1.45-2.12; *P* = .0026) based on 4 studies^2^, ^3^, ^4^, ^5^ comparing 402 patients with previous cancers treated with TNFi, of whom 86 developed new or recurrent cancers to 218 patients with previous cancer treated with anti-α4β7-integrin developing 28 new or recurrent cancers. The IRR was 1.93 (95% CI, 1.85-2.01; *P* = .0002) based on 3 studies^2^, ^3^, ^4^ comparing 2703 PYs of TNFi and 80 new or recurrent cancers to 1396 PYs of anti-α4β7-integrin and 25 new or recurrent cancers (Figures 1 and 2, and Table).

**Table:** Effect Estimates for New or Recurrent Cancer for Biologics vs Biologics Across all Diseases

Outcome characteristics			Biologic group 1			Biologic group 2			Fixed effects		Random effects		Heterogeneity	
Previous cancer	Effect size	Studies, n.	Biologic 1	Sample size/person-years, n.	Cancer events, n.	Biologic 2	Sample size/person-years, n.	Cancer events, n.	Effect size [95% CI]	*P* value	Effect size [95% CI]	*P* value	I^2^ [95% CI], %	Egger's test
Mixed	RR	3	TNFi	218	25	IL-12/23i	56	7	1.00 [0.46; 2.16]	.9988	0.92 [0.16; 5.26]	.8528	2.3% [0.0%; 89.8%]	-
Mixed	RR	4	TNFi	402	86	Anti-α4β7-integrin	218	28	1.75 [1.19; 2.58]	**.0043**	1.75 [1.45; 2.12]	**.0026**	0.0% [0.0%; 84.7%]	-
Mixed	IRR	3	TNFi	2703	80	Anti-α4β7-integrin	1396	25	1.93 [1.23; 3.04]	**.0044**	1.93 [1.85; 2.01]	**.0002**	0.0% [0.0%; 89.6%]	-
Mixed	RR	3	IL-12/23i	56	7	Anti-α4β7-integrin	122	10	1.68 [0.65; 4.38]	.2854	1.64 [0.27; 9.97]	.3601	0.0% [0.0%; 89.6%]	-

*P* values <.05 marked bold.

For TNFi vs IL-12/23i, meta-analysis could only be conducted for the risk of any new or recurrent cancers. The RR was 0.92 (95% CI, 0.16-5.26; *P* = .85) based on 3 studies^2^^,^^4^^,^^5^ comparing 218 patients treated with TNFi developing 25 new or recurrent cancers to 56 patients treated with IL-12/23i developing 7 new or recurrent cancers (Table).

For IL-12/23i vs anti-α4β7-integrin, meta-analysis could only be conducted for the risk of any new or recurrent cancer. The RR was 1.64 (95% CI, 0.27-9.97; *P* = .36) based on 3 studies^2^^,^^4^^,^^5^ comparing 56 patients treated with IL-12/23i developing 7 new or recurrent cancers to 122 patients treated with anti-α4β7-integrin developing 10 new or recurrent cancers (Table).

These results extend previous findings by identifying potential differences in the risk of new or recurrent cancer associated with different biologics.^6^^,^^7^ We found anti-α4β7-integrin to be associated with the decreased risk of new or recurrent cancer when compared with TNFi and numerically but not statistically lower risk when compared with IL-12/23i, though this finding was based on few patients and does not support definitive conclusions regarding differential cancer risk between biologics. Also, a large retrospective study of patients with IBD and no prior cancer found no differences in incident cancers during treatment with either TNFi or vedolizumab.^8^ Moreover, none of the included studies provided adjusted risk estimates, thus emphasizing the necessity of future studies to adjust for cancer-associated risk factors. Interestingly, a recent Danish nationwide study published after the search date of the current study found no substantial differences in the risk of cancer of different biologics in patients with psoriasis.^9^ Notably, we only included patients with IBD, thus highlighting the lack of data regarding other IMIDs like psoriasis and rheumatoid arthritis. Future studies should address the comparative risk of cancer during treatment with biologics in a diverse range of IMIDs in larger cohorts. In conclusion, while potential differences in the risk of new or recurrent cancer between biologics may exist, the available evidence is limited and relies on few patients, and further studies are still needed.

## Supplementary Materials

Supplementary Tables 1 and 2

Extended PDF
