# Supplementary material for: Comparative Risk of Cancer Recurrence During Treatment With Biologics: A Meta-Analysis
Source: Gastro Hep Adv. 2026 Aug 15;5(12):101092. doi: 10.1016/j.gastha.2026.101092 (PMC13625878; doi:10.1016/j.gastha.2026.101092)
Supplement: Supplementary file 1 [file mmc1.pdf]

Supplementary Tables

Supplementary table 1: General characteristics of the included studies

**Study information**					**Biologics treatment group**					**Control group**					**Risk assessment**	
Study	Country	Population source (Study period)	Study design	Initial cancer type (Follow-up, years)	Type of biologic and patients receiving therapy, no.	No. of patients (Cases of new or recurrent cancer)	Age (years), mean ± SD	Male, no. (%)	Patient-years, no.	Control drug	No. of controls (Cases of new or recurrent cancer)	Age (years), mean ± SD	Male no. (%)	Patient-years, no.	Risk	Adjustment
**Inflammatory Bowel Disease**																
Hasan (2022)[2]	USA	8 Cleveland Clinic centers (January 2014 – January 2020)	Retrospective cohort study	Mixed (Median: 5.2)	VDZ: 34	34 (1)	39±18	21 (62)	266	TNFi	99 (7)	36±17	44 (44)	938	NA	NA
					UST: 27	27 (3)	38±17	21 (62)	164							
Vedamurthy (2022)[3]	USA	Partners' Healthcare system (2014-2019)	Retrospective cohort study	Mixed (Median: 6.2)	TNFi: 184	184 (61)	40±18	97 (53)	1452	VDZ	96 (18)	40±18	43 (45)	821	NA	NA
Hong (2022)[4]	USA	Academic medical center (January 2013 – December 2020)	Retrospective cohort study	Mixed (Median: 4.33)	VDZ: 37	37 (6)	42.5±18.6	21 (56.8)	309	TNFi	41 (12)	43.5±20.3	24 (58.5)	313	NA	NA
					UST: 14	14 (2)	46.8±18.4	8 (57.1)	67							
Holmer (2023)[5]	USA	Multicenter (- December 31, 2020)	Retrospective cohort study	Mixed (NA)	UST: 15	15 (2)	53.3±14.5 *	34 (51.5) *	UST	TNFi	78 (6)	51.8±14.2	36 (46.2)	NA	NA	None
					VDZ: 51	51 (3)	53.3±14.5 *	34 (51.5) *	NA							

Abbreviations:
Tumor Necrosis Factor-α inhibitor (TNFi); Vedolizumab (VDZ); Ustekinumab (UST); * Collectively for non-TNFi biologics (VDZ+UST)

**Supplementary table 2:** Effect estimates for recurrent cancer for biologics vs biologics across all diseases													
			**Biologic group 1**			**Biologic group 2**			**Fixed effects**		**Random effects**		**Heterogeneity**	
**Previous cancer**	**Effect size**	**Studies, n.**	**Biologic 1**	**Sample size/person-years, n.**	**Cancer events, n.**	**Biologic 2**	**Sample size/person-years, n.**	**Cancer events, n.**	**Effect size [95% CI]**	**P-value**	**Effect size [95% CI]**	**P-value**	**I² [95% CI], %**	**Egger's test**
**Recurrent cancer**														
Mixed	RR	3	TNFi	324	38	anti-α4β7-integrin	167	14	1.51 [0.85; 2.71]	0.1626	1.47 [0.77; 2.78]	0.1245	0.0% [0.0%; 89.6%]	-
Mixed	IRR	3	TNFi	2703	38	anti-α4β7-integrin	1396	14	1.64 [0.88; 3.06]	0.1199	1.56 [0.77; 3.19]	0.114	0.0% [0.0%; 89.6%]	-
**New cancer**														
Mixed	RR	3	TNFi	324	42	anti-α4β7-integrin	167	11	2.18 [1.15; 4.10]	**0.0163**	2.18 [1.47; 3.24]	**0.0136**	0.0% [0.0%; 89.6%]	-
Mixed	IRR	3	TNFi	2703	42	anti-α4β7-integrin	1396	11	2.30 [1.18; 4.47]	**0.0139**	2.30 [1.39; 3.81]	**0.0191**	0.0% [0.0%; 89.6%]	-

Abbreviations: Tumor Necrosis Factor-α inhibitor (TNFi); Risk Ratio (RR), Incidence Rate Ratio (IRR),
p-values <0.05 marked bold.
